# Ubiquitination by HUWE1 in tumorigenesis and beyond

**DOI:** 10.1186/s12929-018-0470-0

**Published:** 2018-09-04

**Authors:** Shih-Han Kao, Han-Tsang Wu, Kou-Juey Wu

**Affiliations:** 1 Research Center for Tumor Medical Science, China Medical University, No. 91, Hseuh-Shih Rd, Taichung, 40402 Taiwan; 2 Drug Development Center, China Medical University, Taichung, 40402 Taiwan; 3 Institute of New Drug Development, Taichung, 40402 Taiwan; 4 Graduate Institutes of Biomedical Sciences, China Medical University, Taichung, 40402 Taiwan; 5 Departmet of Medical Research, China Medical University Hospital, Taichung, 40402 Taiwan; 6 Department of Cell and Tissue Engineering, Changhua Christian Hospital, Changhua City, 500 Taiwan

**Keywords:** Ubiquitination, HUWE1, Tumorigenesis, Cancer therapeutics

## Abstract

Ubiquitination modulates a large repertoire of cellular functions and thus, dysregulation of the ubiquitin system results in multiple human diseases, including cancer. Ubiquitination requires an E3 ligase, which is responsible for substrate recognition and conferring specificity to ubiquitination. HUWE1 is a multifaceted HECT domain-containing ubiquitin E3 ligase, which catalyzes both mono-ubiquitination and K6-, K48- and K63-linked poly-ubiquitination of its substrates. Many of the substrates of HUWE1 play a crucial role in maintaining the homeostasis of cellular development. Not surprisingly, dysregulation of HUWE1 is associated with tumorigenesis and metastasis. HUWE1 is frequently overexpressed in solid tumors, but can be downregulated in brain tumors, suggesting that HUWE1 may possess differing cell-specific functions depending on the downstream targets of HUWE1. This review introduces some important discoveries of the HUWE1 substrates, including those controlling proliferation and differentiation, apoptosis, DNA repair, and responses to stress. In addition, we review the signaling pathways HUWE1 participates in and obstacles to the identification of HUWE1 substrates. We also discuss up-to-date potential therapeutic designs using small molecules or ubiquitin variants (UbV) against the HUWE1 activity. These molecular advances provide a translational platform for future bench-to-bed studies. HUWE1 is a critical ubiquitination modulator during the tumor progression and may serve as a possible therapeutic target for cancer treatment.

## Background

Amongst various post-translational modifications, ubiquitination is a common yet significant process in cells. Discovered in the 1980s by Goldstein [1], ubiquitination has since been modeled as a process that marks proteins for degradation [2, 3]. However, a growing body of evidence has shown that ubiquitination modulates disparate functions other than proteolysis, such as protein trafficking, signaling transduction, enzymatic activities, chromatin structure, nuclear localization, genome integrity [4–8]. Therefore, dysregulation of the ubiquitin system can lead to pathogenesis, including tumor development.

In this review, we briefly introduce the process of ubiquitination and the structure of HUWE1. Then, we address some of the substrates of HUWE1-mediated ubiquitination and their signaling and functional alterations in tumorigenesis and beyond. Finally, we summarize the clinical relevance, the underlying challenges of expanding the atlas of HUWE1 substrates and the possible application of HUWE1 in cancer therapeutics.

## The process of ubiquitination

The attachment of ubiquitin (8.5 kDa) to a substrate protein requires the actions of three distinct enzymes and steps. The first step activates ubiquitin by forming a thioester linkage with ubiquitin-activating enzyme, E1. Subsequently, ubiquitin is transferred to an ubiquitin-conjugating enzyme, E2. Finally, an E3 ligase catalyzes the bonding between ubiquitin and a lysine residue of a target protein. The E3 ligases are responsible for substrate recognition and render substrate specificity. Therefore, E3 ligases are considered the most important components in this ubiquitin machinery. Ubiquitin ligases fall into three classes based on their structural domains: 1) the homologs to the E6-AP carboxyl terminus (HECT) domain, 2) the really interesting new gene (RING) domain, and 3) the U-box domain. While other E3 ligases act as adaptors to bring charged E2s close to substrates to facilitate ubiquitination, HECT-domain E3s form a covalent thioester intermediate with ubiquitin during ubiquitination [9]. Many E3 ligases have been implicated in tumorigenesis because most of their known substrates are oncogenes or tumor suppressors. For instance, the Skp1/Cul1/F-box (SCF) complex represents a family of the multi-protein E3 ubiquitin ligase complex, which catalyzes ubiquitination of proteins and controls various cellular events, especially the cell cycle, by proteasomal degradation [10]. Dysregulation of the cell-cycle ubiquitin-proteasome system can result in perturbed cell growth and enhanced tumorigenesis. The F-box protein, Skp2, is the E3 ligase in the SCF complex responsible for substrate recognition and promotes the degradation of p27 Cdk inhibitor during S-phase [11]. Excessive amount of Skp2 results in a loss of p27 and this oncogenic role of Skp2, corresponding to up-regulation of Skp2 in tumor tissues, has been observed in a wide range of human cancers, including lung, breast, colorectal, head and neck squamous cell carcinoma (HNSCC) [12–14]. Inhibition of Skp2 has been demonstrated to potentially restrict cancer progression [12, 13]. On the contrary, the F-box protein, FBW7, is considered a tumor suppressor, as it degrades proto-oncogenes, such as Myc, cyclin E, Jun, Mcl-1, and Notch [14–19]. Therefore, understanding the modulation and the molecular mechanism of E3 ligases is of paramount importance in understanding tumor development and progression.

Interestingly, ubiquitin can be conjugated to a substrate in more than one form and different types of ubiquitination control diverse biological consequences [20]. The processes by which proteins are ubiquitinated on a single lysine residue by a single ubiquitin is called mono-ubiquitination. This modification has been found to regulate receptor internalization, degradation in lysosomes and protein recycling [21]. Aside from mono-ubiquitination, other well-established types of modification are K48-linked and K63-linked poly-ubiquitination. The conjugation of poly-ubiquitination is achieved via isopeptide bonds between the carboxylate at the C-terminal glycine of the distal ubiquitin and the ε–amine of an internal lysine of the proximal ubiquitin [22]. Whereas K48-linked polyubiquitin target proteins for proteasomal degradation, K63-linked polyubiquitin chains are involved in a wider variety of regulations, including signal transduction, protein localization, DNA repair, endocytosis, and protein-protein interaction [23–25]. Other types of ubiquitin linkage have been discovered as well, such as K6, K11, K27, K29, and K33 [26], increasing the versatility and the functional repertoire of ubiquitination.

## Structure of HUWE1

**Fig. 1:**
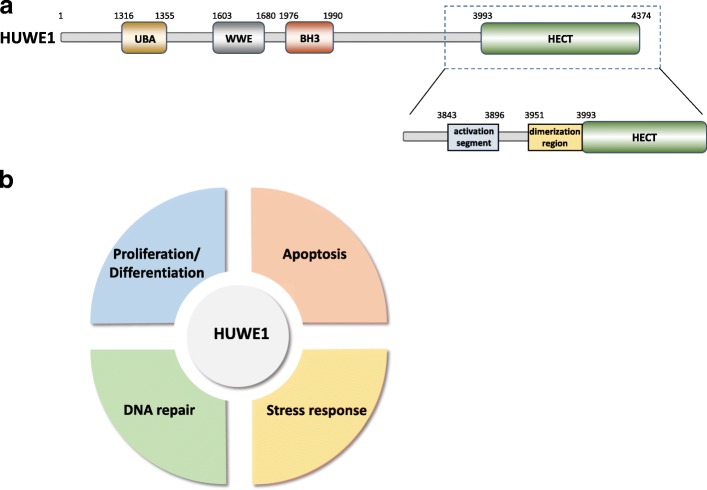
The functional domains of HUWE1 and functional enrichment of HUWE1. **a** Schematic diagram of the structural features of HUWE1 protein. Huwe1 protein mainly contains four domains: 1) the UBA domain and 2) the WWE domain, 3) the BH3 domain, and 4) the HECT domain. The HECT domain contains a catalytic cysteine residue for the ubiquitin-thioester bond formation and therefore carries the enzymatic activity of HUWE1 protein. Crystal structural analysis shows that the activity of HUWE1 can be self-activated through an activation segment. HUWE1 proteins exhibit the dimerization ability using the region close to the N-terminus of the HECT domain. **b** Functional enrichment of HUWE1. Based on the substrates regulated by HUWE1, HUWE1 is involved in four major aspects of cellular regulation: Proliferation/differentiation, apoptosis, DNA repair, and stress response. Dysregulation of HUWE1, either through the impaired enzymatic activity or the aberrant expression of HUWE1, leads to disease development, such as tumor formation

The HECT domain-containing ubiquitin E3 ligase, HUWE1 (also known as HectH9, ARF-BP1, URE-B1, Mule, and LASU1), was first detected from a size-fractionated human brain cDNA library by Nagase et al. [27], and the full-length human HUWE1 was then identified by Liu et al., which they termed LASU1 [28]. Encoded by the *HUWE1* gene, the 482-kDa-sized HUWE1 contains two N-terminal domains, DUF908 and DUF913 (*d*omains of *u*nknown *f*unctions), similar to domains in an *S. cerevisiae* HECT ligase, Tom1, followed by a ubiquitin-associated (UBA) domain [28]. A WWE domain involved in the regulation of ubiquitin-dependent proteolysis [29] and a BH3 domain shared by all the Bcl-2 family members are also contained within HUWE1 [30]. At the C-terminus of HUWE1, there is a HECT domain which contains a catalytic cysteine residue for ubiquitin-thioester formation, and thus the HECT domain carries the enzymatic activity of this protein [31] (Fig. 1a). Structural studies have shown that the canonical HECT domain is composed of a bilobal architecture (the N-terminal (N) lobe and C-terminal (C) lobe) that separates the E2 binding region and the catalytic cysteine [32–34]. The N lobe of HECT is composed of 13 α–helices and 7 β–strands and the C lobe is comprised of 4 α–helices and 4 β–strands [31]. Crystal structural analysis of the HECT domain of HUWE1 reveals that helix α1 in the N lobe stabilizes the HECT domain and modulates the autoubiquitination ability of HUWE1 by reducing the rate of Ub addition to the HECT domain [31]. According to Pandya et al., removal of helix α1 may cause a more relaxed form of HUWE1 that exhibits greater intradomain flexibility, possibly leading to the increased enzymatic activity [31].

Highly conserved in mammals, the amino acid sequences of human and mouse HUWE1 are more than 90% identical [30], and expression of *HUWE1* is high in a considerable amount of mouse tissues, including cortex, hippocampus, eye, tongue, liver, kidney, adrenal gland, and fibroblasts [35]. Its ubiquitous expression suggests that HUWE1 may participate in quite a number of cellular functions. More importantly, while most HECT domain-containing E3s synthesize polyubiquitin chains with linkage preferences, HUWE1 is one of the few HECT E3s that can mediate both K48- and K63-linked ubiquitination [36–38]. Notably, HUWE1 can also assemble mono-ubiquitination on its substrates and the less-known K6-linked poly-ubiquitination [39–42], suggesting HUWE1 plays multiple regulatory roles via mono−/poly-ubiquitination and linkage diversities.

By functional enrichment, HUWE1 is highly associated with proliferation/differentiation, apoptosis, DNA repair, and stress response (Fig. 1b), but the impact of HUWE1 in other aspects of functions has been studied. In the next section, we introduce the substrates of HUWE1 and the signals that modulate HUWE1.

## Substrates of HUWE1

### MYC

**Fig. 2:**
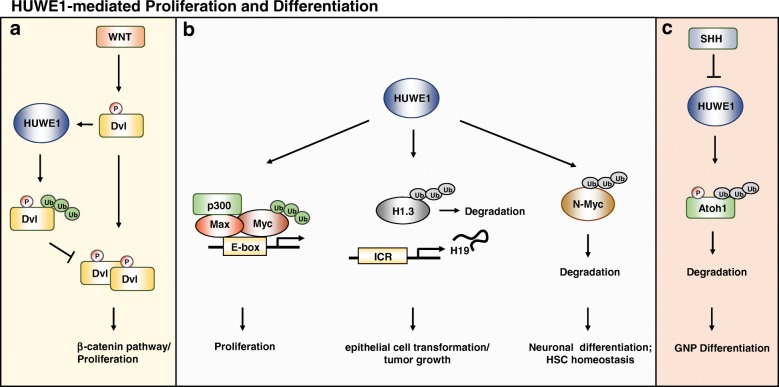
HUWE1-mediated proliferation/differentiation. **a** HUWE1 is regulated by WNT and ubiquitinates Dvl via K63-linkage, which subsequently inhibits multimerization of Dvls, contributing to the negative feedback loop in the Wnt/β-catenin pathway. **b** HUWE1 is able to enhance tumor proliferation by K63-ubiquitinating c-Myc, which facilitates the recruitment of CBP/p300, therefore enhancing the transactivation activity of c-Myc. HUWE1 sustains normal ovarian epithelial cell transformation and tumor growth by ubiquitinating histone H1.3. Ubiquitinated H1.3 is subsequently degraded, releasing H1.3 from the imprinting control region (ICR) of the distal promoter region of an oncogenic non-coding RNA, H19. HUWE1 also mediates K48-linked polyubiquitination of N-myc and facilitates its degradation. Loss of N-myc can arrest proliferation via cell cycle and begin differentiation in neural stem/progenitor cells. Loss of N-myc by HUWE1 can also disrupt the neural stem cell activity through the DLL3-Notch pathway in the mouse cortex. In hematopoietic stem cells (HSCs), loss of HUWE1 increases N-myc-dependent proliferation and thus HUWE1 is a key regulator in the maintenance and lymphoid commitment of HSCs. **c** HUWE1 regulates SHH-type medulloblastoma (MB) via ubiquitinating and controlling Atoh1 protein turnover. Atoh1, a crucial basic helix-loop-helix transcription factor for granule neuron progenitors (GNPs), inhibits neuronal differentiation and enhances MB formation

Myc was one of the first proteins discovered to be a substrate of HUWE1. This proto-oncogene can act as a transcription activator or repressor, depending on the components in the transcription complex. In the binary module of the complex, Myc, along with Max, binds to a specific DNA sequence, the E-box element (CACGTG), and activates transcription of a set of pro-growth genes [43]. However, when Myc forms a ternary complex with Max and Miz1, it represses transcription through interruption of the interaction between Miz1 and p300 histone acetyltransferase [44] and by recruiting a DNA methyltransferase, DNMT3a [45]. Increased levels of Myc in cells lead to cell growth, transformation, and tumorigenesis. One of the hallmarks of Myc-mediated cell transformation is the elevated activity of cyclin D-CDK4. On one hand, Myc transactivates CDK4, causing G1 progression in response to mitogenic signals [46]. On the other, genes of INK inhibitors (p15^INK4b^ and p18^INK4c^) and CIP1/KIP1 (p21 and p27) are repressed by Myc, leading to enhanced cyclin D-CDK4 activity [47–49]. As a short-lived protein, Myc is known to be ubiquitinated by Skp2, FBW7, FBXO28, FBXL14 [50–53]. Ubiquitination of Myc by HUWE1, however, is far more complicated. In one model, c-Myc is K63-linked polyubiquitinated by HUWE1 at the C-terminus, which is required for recruitment of the coactivators, p300 [43] (Fig. 2). In this model, site-specific ubiquitination dictates the switch between transcription activation and repression of Myc. Knockdown of HUWE1 reduces Myc functions in breast cancer cell lines and co-depletion of HUWE1 and Mnt, an antagonist of transactivation by Myc further abolished the colony formation in HeLa cells [54], suggesting an oncogenic role of HUWE1 in regulating Myc functions.

The related N-Myc, normally expressed in neural stem cells and neuroectodermal progenitors, needs timely withdrawal to initiate differentiation during neural development [55]. Premature disappearance of N-Myc results in precocious differentiation and disturbed cell cycle and N-Myc expression plays a key role during neuroblastoma differentiation [56, 57]. To ensure accurate expression of N-Myc, N-Myc protein degradation by the ubiquitin-proteasome system is essential to control the temporal expression of N-Myc [58, 59]. Liquid chromatography-tandem mass spectrometry (LC-MS/MS) results have identified that the HUWE1 is associated with N-Myc in neural cells and HUWE1-mediated N-Myc ubiquitination directs N-Myc degradation via K48-linkage assembly [60]. *Cyclin D2*, a downstream target gene of N-Myc, is decreased in N-Myc knockdown cells but increased in HUWE1-silenced cortices [60], demonstrating that this HUWE1-N-Myc pathway controls neural differentiation of mouse ES cells [60]. A follow-up study showed that the DLL3-Notch pathway is excessively activated by N-Myc accumulation in the HUWE1-knockout mouse brain, leading to defected neurogenesis [61]. Zhao et al. have further demonstrated that human high-grade gliomas contain focal hemizygous deletions of the X-linked *HUWE1* gene and amplification of the *N-myc* locus [61]. HUWE1 also plays a role in hematopoietic stem cells (HSC) self-renewal, quiescence and lymphoid fate specification by regulating the expression of N-Myc [62]. Upregulation of N-Myc increased proliferation and stem cell exhaustion in HUWE1-knockout HSCs [62]. Together these data show that HUWE1 is important in both the development of neurogenesis and maintenance of HSC homeostasis by tightly controlling N-Myc expression (Fig. 2).

Contradictory to the observation that c-Myc forms polyubiquitin chains via K63 linkage [43], Zhao et al. have shown that both N-Myc and c-Myc can be ubiquitinated by HUWE1 through K48-mediated linkage and genetic knockout of *HUWE1* in embryonic stem (ES) cells can stabilize both N-Myc and c-Myc [60]. In their study, Zhao et al. used a truncated HUWE1, where c-Myc binds to HUWE1 less efficiently than N-Myc [60], which might be the reasons why contrasting observations are presented by different groups. It is thus possible that c-Myc can be tagged with K48- and K63-linked ubiquitin moieties in vivo by HUWE1. This difference may emblem diverse biological influences in time- or context-dependent regulations of Myc by HUWE1.

### Dvl

It has recently been verified that HUWE1 regulates Dishevelled (Dvl) multimerization in the Wnt signaling pathway [63]. HUWE1 promotes K63-linked poly-ubiquitination of Dvl at the DIX domain, inhibiting Dvl multimerization. Importantly, the interaction between HUWE1 and Dvl depends on Wnt3a stimulation or CK1 phosphorylation, and this leads to a negative feedback loop to inhibit Wnt signaling [63] (Fig. 2).

### Atoh1

Medulloblastoma (MB) is the most common malignant pediatric brain tumor with a 20–30% incidence rate among all central nervous system (CNS) malignancies and an overall 5-year survival rate of 70–80% [64, 65]. Disruptions in cerebellar development have been shown to cause medulloblastoma [66]. Recent molecular analyses have divided medulloblastoma into four subgroups (WNT, SHH, Group 3, and Group 4) whose genetic differences lead to distinct prognoses, temporal, and anatomical patterns of recurrence [67, 68]. Associated with the development of cerebellum, knockout of HUWE1 increases the proliferation of cerebellar granule cells in the external granule layer (EGL) of postnatal cerebella and results in disorganization of glial cells and granule neuron migration defects [69]. HUWE1 plays a significant role in Group 2 sonic hedgehog (SHH)-type of medulloblastoma by regulating the turnover of the basic helix-loop-helix (bHLH) transcription factor, Atoh1, which prevents differentiation of cerebellar granule neuron progenitors (GNPs) and enhances medulloblastoma (MB) formation [66, 70]. Upon SHH treatment, Atoh1 is stabilized, which can transform GNPs into MB-initiating cells by suppressing neuronal differentiation [70]. Conversely, when the ligand, SHH, is subtracted, HUWE1 is immediately recruited to facilitate Atoh1 ubiquitination and degradation in a phosphorylation-dependent manner [71] (Fig. 2). The high HUWE1 mRNA level correlates with a better survival in patients with SHH medulloblastoma and this prognostic role of HUWE1 may serve as a platform for a combination therapy for SHH medulloblastoma where both SHH signaling and Atoh1 via the HUWE1 pathway are inhibited [71].

### Miz1

**Fig. 3:**
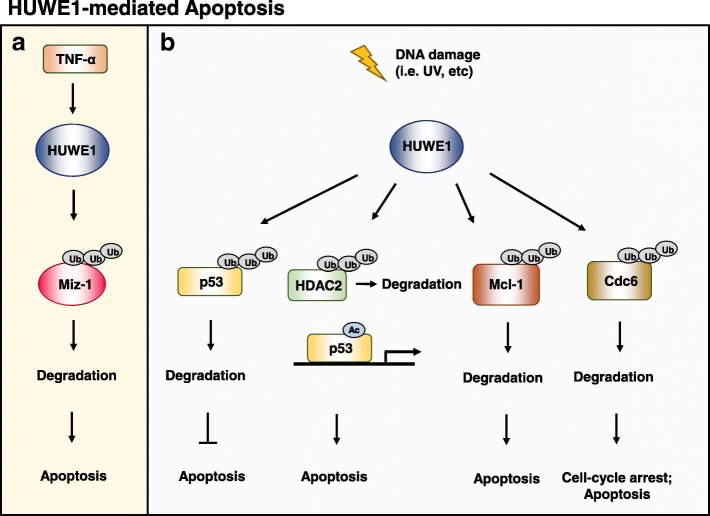
HUWE1-mediated apoptosis. **a** Upon TNFα stimulation, HUWE1 can mediate K48-linked polyubiquitination of Miz-1, thereby facilitating the degradation of Miz-1 protein. Miz-1 suppresses TNFα-induced JNK activation and cell death. Reduced Miz-1 levels relieve this negative regulation on TNFα. **b** When DNA damage occurs, cells undergo cell-cycle arrest and apoptosis by enhancing the activities of p53, downregulating the expressions of anti-apoptotic molecules (i.e. Mcl-1) and the assembly of pre-replicative complex (Cdc6 in preRC). HUWE1 is an associating protein of the tumor suppressor, ARF, which plays a pivotal role in regulating p53 and inhibits the ubiquitin ligase activity of HUWE1. Expression of HUWE1 can directly ubiquitinate and degrade p53 in a Mdm2-independent way, thereby suppressing p53-dependent apoptosis. HUWE1 mediates polyubiquitination of HDAC2, which deacetylates p53 and affects p53 transcriptional activity. HUWE1 deficiency leads to accumulation of HDAC2 and compromised p53 acetylation and apoptotic response upon DNA damage. HUWE1 interacts with Mcl-1 through the BH3 domain, causing Mcl-1 degradation upon DNA damage. Cdc6 plays a key role in DNA replication and degradation of Cdc6 mediated by HUWE1 polyubiquitination occurs upon ultraviolet irradiation or DNA alkylation, resulting in cell-cycle arrest and apoptosis

Miz1 is a zinc-finger transcription factor which binds to the core promoter of several genes [72, 73] and regulates their expressions, including Bcl-2 [74], p15^INK4b^ [48], p21^CIP1^ [47]. Miz-1 is a negative regulator of tumor necrosis factor alpha (TNFα) signaling [75, 76]. Yang et al. have shown that upon TNFα stimulation, HUWE1 degrades Miz-1 protein and facilitates TNFα-induced JNK activation [77] (Fig. 3). Knockdown of HUWE1 restrained TNFα-induced JNK activation and cell death but the effect was abolished in Miz1^−/−^ MEFs [77]. In another *HUWE1* knockout mouse model, HUWE1 deletion causes increased severity of skin tumors, which were induced by carcinogens, 7,12-dimethylbenz-(a)-anthracene (DMBA) and 12-O-tetradecanoylphorbol-13-acetate (PMA) [78]. HUWE1 deficiency results in an accumulation of the c-Myc/Miz1 complex and concomitant knockout of c-Myc could rescue HUWE1-deficient phenotype [78]. In vitro knockdown of MiZ1 could reverse and reduce proliferation of HUWE1-deficient keratinocytes. Increased c-myc and Miz1 by HUWE1 depletion leads to a repression of *Cdkn2b* (p15^INK4b^) and *Cdkn1a* (p21^CIP1^), thereby enhancing tumorigenesis [78].

### p53

Tumor suppressor p53 is a crucial coordinator in cellular responses to stress from oncogene activation, DNA damage, and hypoxia [79, 80]. The antiproliferative capability of p53 mediates cell cycle arrest, apoptosis, and even cellular senescence [81, 82]. While mutations of p53 are observed in more than half of human tumors [83], dysregulation of the ARF tumor-suppressor protein (p14^ARF^ in humans and p19^ARF^ in mouse), which increases and activates wild-type p53 by sequestering Mdm2 in the nucleolus [84] or by directly inhibiting the enzymatic activity of Mdm2 [85, 86], is a common characteristic in cancer as well. Chen et al. have identified HUWE1 (termed ARF-BP1 in their report) as a major associating protein of ARF in p53-null cells and ARF induces p53-independent growth suppression by inhibiting the ubiquitin ligase activity of HUWE1 [87]. In p53-wildtype cells, however, HUWE1 directly binds to and ubiquitinates p53, which facilitates p53 protein degradation and subsequently suppresses p53-dependent apoptosis [87] (Fig. 3). ARF induces p53 stabilization through negatively regulating HUWE1, which is independent of Mdm2 [87], suggesting that multiple E3 ligases are involved in regulating p53 turnover. Dysregulation of p53 ubiquitination is thus a pivotal mechanism in tumorigenesis. Recently, reports have shown that suppression of HUWE1 elevates the p53 protein levels in Myc-driven B cell lymphomas, leading to growth arrest and apoptosis [88]. Together these findings favor the notion that HUWE1 promotes tumorigenesis and may serve as a therapeutic target as a future research direction.

### HDAC2

Histone deacetylases (HDACs) mediates a broad range of cellular functions by epigenetically modulating chromatin structure and gene transcription. While acetylation is mediated by histone acetyltransferase (HAT) and is thought to activate gene transcription, HDACs reverses this process and mediates transcriptional suppression [89]. There are four major classes of HDACs. Class I HDACs are HDAC1–3, and HDAC8, which are ubiquitously present in cells and possess the strongest HDAC activities. Thus, both histone and non-histone proteins can be the substrates of Class I HDACs. Class II HDACs are HDAC4–7 and HDAC9 and their expressions are tissue specific. Class III HDACs, often referred to as sirtuins, require NAD^+^ as a cofactor and are homologous with yeast Sir2. The Class VI HDAC (HDAC11) is homologous with Class I and Class II HDACs, all of which are Zn^2+^-dependent enzymes as opposed to Class III HDACs [90, 91].

Investigation of HUWE1 and its role in DNA damage using HDAC inhibitors (HDACis) has shown that HUWE1 specifically ubiquitinates HDAC2 and negatively regulates its stability [92]. In *HUWE1* knockout embryonic fibroblasts (MEFs), increased HDAC2 abrogates acetylation of p53, a critical modification for p53 transcriptional activity, and apoptosis is attenuated under cisplatin or the HDACi, NaBu, treatments [92]. Collectively, these data show that the HUWE1-HDAC2 pathway controls cell apoptosis via modulating the transcriptional activity of p53 (Fig. 3).

### Mcl-1

In the transcription-independent pathway of apoptosis, the stress signal induces mitochondrial outer membrane permeabilization (MOMP) and prompts the release of proapoptotic factors of Bcl-2 homology (BH)3-only molecules, such as BAD, BID, BIM, PUMA, and NOXA while the anti-apoptotic members of the Bcl-2 family antagonizes this event [93–95]. Mcl-1 is an anti-apoptotic member of the Bcl-2 family proteins which is frequently upregulated in cancers to promote cell survival [96–99]. Under normal conditions, Mcl-1 associates with the proapoptotic BAK protein to maintain BAK in an inactive state whereas downregulation of Mcl-1 proteins is triggered by DNA damage, such as infection by viruses [100]. HUWE1 contains a BH3 domain for specific interaction with Mcl-1 and facilitates the degradation Mcl-1, thereby enhancing apoptosis in response to DNA damage [30] (Fig. 3). The well-conserved BH3 domain within HUWE1 mostly resembles that of Bak and therefore HUWE1 might compete with Bak to break up the Mcl-1/Bak complex, converting the fate of a cell toward death. Further validation is needed to confirm the relationship among Bak, HUWE1, and Mcl-1 in the mitochondria-mediated apoptotic pathway.

Regulation of HUWE1-induced Mcl-1 degradation, however, is not limited to DNA damage or viral infection. During obesity-associated hepatocellular carcinoma (HCC) development, expression of interleukin-6 (IL-6), which results from a chronic low-grade proinflammatory state in white adipose tissue and liver [101], helps to stabilize Mcl-1 via promotion of GSK3β inactivation and suppression of HUWE1 [102]. As a result, obesity suppresses hepatocyte apoptosis through Mcl-1 stabilization and promotes liver carcinogenesis [102]. Whether HUWE1 also mediates Mcl-1 degradation in other cell types, as an alternative major regulatory pathway to control tumorigenesis, remains to be determined.

### Cdc6

DNA damage can influence cell cycle by not only promoting checkpoint functions but reducing re-replicating DNA as well to block mitosis of cells with erroneous DNA. The prereplication complex (preRC) is assembled during G1 to facilitate replication and Cdc6 is an essential regulator in this complex [103]. In later cell cycle stages, Cdc6 takes part in checkpoint activation if DNA replication is not properly completed [104–106]. Cdc6 proteins are rapidly degraded during each cell cycle by the E3 ligase, anaphase promoting complex (APC^Cdh1^) [107]. Upon DNA damage, Cdc6 is also ubiquitinated and degraded by APC^Cdh1^ in a p53-dependent manner [108]. However, Cdc6 degradation can occur independently of both p53 and cell cycle when DNA damage is caused by ultraviolet (UV) irradiation or DNA alkylation by methyl methane sulfonate (MMS) and this poly-ubiquitination process is mediated by HUWE1 [103] (Fig. 3). As a result, Cdc6 is directed to the proteasomal degradation route and released from chromatin. It is tempting to postulate that in response to DNA damage, HUWE1 mediates Cdc6 degradation to inhibit new preRC assembly, but may keep functional acetylated p53 in abundance to push cells to undergo cycle arrest, and finally apoptosis.

### BRCA1

**Fig. 4:**
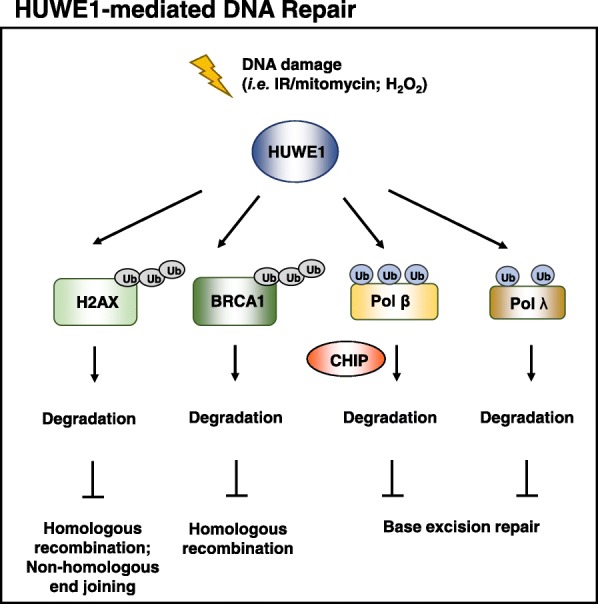
HUWE1-mediated DNA repair. HUWE1 mediates H2AX and BRCA1 ubiquitination and degradation. Upon double-strand breaks (DSB), two major DNA repair systems are activated: homologous recombination (HR) and non-homologous end joining (NHEJ). Phosphorylation of H2AX (γH2AX) initiates a DNA damage signaling cascade and has been implicated in both DSB repair systems. BRCA1 is a pleiotropic DNA damage response protein and mainly plays a significant role in HR. HUWE1 also participates in base excision repair by regulating the protein turnover of Pol β, and Pol λ. HUWE1 catalyzes mono-ubiquitination of Pol β at Lys-41, 61, and 81 and of Pol λ at Lys-27 (major site) and Lys-273. Mono-ubiquitinated Pol β is later poly-ubiquitinated by another E3 ligase, CHIP, for protein degradation. Pol λ mono-ubiquitination, on the other hand, is regulated by Cdk2/cyclinA-mediated phosphorylation. As ARF negatively regulates the HUWE1 activity, knockdown of ARF decreases the protein levels of Pol β and Pol λ

Loss of BRCA1 is a common characteristic in breast and ovarian cancers and causes defects in DNA repair, especially double-strand breaks (DSBs) by homologous recombination (HR), resulting in genomic instability [109–111]. Although germline mutations of BRCA1 are a risk factor of hereditary breast and ovarian cancers among women, their occurrence is less frequent in sporadic cancers [112]. Instead, a decrease in BRCA1 protein levels is more commonly detected, implying that a deregulation of the machinery of proteasomal degradation may take part in BRCA1-mediated tumorigenesis. Enzymes that regulate the turnover of BRCA1 have recently been identified, including HERC2, SCF^FBXO44^ as the E3 ligases [113, 114], and E2T, as an ubiquitin-conjugating E2 enzyme [115]. It has been shown that HUWE1 associates with the BRCA1-Merit40/RAP80 complex [116] and together mediate the ubiquitination and degradation of BRCA1 (Fig. 4). Upon ionizing radiation or mitomycin treatment, knockdown of HUWE1 confers treatment resistance to breast cancer cells [117]. The antagonistic effect of HUWE1 on BRCA1 may suppress HR-dependent DSB repair, supporting the role of HUWE1 in DNA damage repair.

### Polβ & Polλ

HUWE1 also participates in DNA-damage response as a tuning mechanism for base excision repair (BER), which requires a set of enzymes to recognize and remove DNA lesions induced by DNA damaging agents and restore the correct DNA information. BER DNA polymerases are enzymes filling in the one nucleotide gap to replace the excised one during BER. Pol β is the major human polymerase that catalyzes this gap-filling process; therefore, its under- or over-expression may lead to severe mutagenesis and cancer susceptibility [118, 119]. In the absence of Pol β, however, Pol λ can compensate the functions of Pol β as both proteins share noteworthy enzymatic and structural similarities [120]. Interestingly, both Pol β and Pol λ can be ubiquitinated by HUWE1 and another E3 ligase, carboxyl terminus of Hsc70 interacting protein (CHIP) [39, 121].

In the case of Pol β, Parsons et al. observed that HUWE1 and ARF oppositely modulate the BER activity by controlling the stability of Pol β [39]. HUWE1 first catalyzes mono-ubiquitination at Lys-41, 61, and 81 of Pol β, which is subjected to CHIP-mediated poly-ubiquitination and subsequent degradation [39]. Double silencing of HUWE1 and CHIP only marginally elevates Pol β protein expression, suggesting that these two E3 ligases orchestrate Pol β degradation in the same pathway. Knockdown of ARF, which is known to inhibit the ubiquitination activity of HUWE1 [87], however, could increase the levels of mono-ubiquitinated Pol β [39]. While ARF knockdown slows down the rate of DNA repair, HUWE1 silencing conspicuously increases the repair efficacy of hydrogen peroxide-induced DNA lesions because of the enrichment of Pol β in the nucleus [39]. Hence, HUWE1, ARF and CHIP together modulate BER capacity by controlling the protein dynamics of Pol β [39] (Fig. 4). Similar to Pol β, ARF also modulates the steady state of Pol λ as knockdown of ARF decreases the protein levels of Pol λ [40]. HUWE1 ubiquitinates Pol λ at Lys-27 (major site) and Lys-273 (minor site), but unlike Pol β, a subsequent in vivo regulation by CHIP has not been further validated (Fig. 4). Nevertheless, ubiquitination of Pol λ is regulated by Cdk2/cyclinA-mediated phosphorylation [40]. Phosphorylation of Pol λ inhibits protein degradation by keeping Pol λ proteins on chromatin in the nucleus [40]. Increased ubiquitination and degradation by HUWE1 is observed when phosphorylation-deficient mutant of Pol λ is expressed, resulting in error-prone single-nucleotide incorporation upon 8-oxo-G DNA treatment [40]. It remains to be answered what stimulus is involved in activating each repair Pol and whether post-translational modifications (PTMs), such as phosphorylation, would contribute to Pol subcellular localization and degradation.

### HAUSP (USP7)

**Fig. 5:**
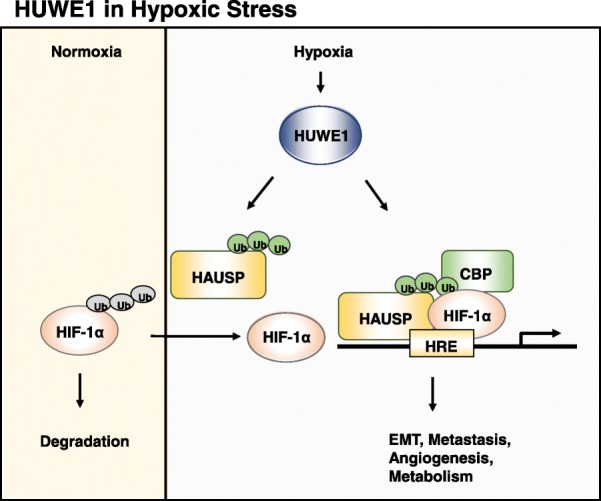
HUWE1 under hypoxic stress. HIF1α is a labile protein under noromoxia but the HIF1α protein is stabilized under hypoxia via HUWE1. The deubiquitinase, HAUSP, is K63-polyubiquitinated by HUWE1 under hypoxia. K63-polyubiquitinated HAUSP has the enhanced deubiquitinase activity to stabilize HIF-1α. Moreover, K63-polyubiquitinated HAUSP anchors HIF-1α, CBP, the mediator complex, and the super-elongation complex to enhance the gene transcription activity initiated by HIF-1α, thereby increasing hypoxia-associated tumor aggressiveness

HAUSP (USP7) is a USP type deubiquitinase that stabilizes many proteins via deubiquitination. Amongst its substrates, MDM2 and PTEN have been shown to be deubiquitinated by HAUSP to cause the stabilization or change of cellular localization, respectively, leading to tumorigenesis [122, 123]. Recent results have also shown that HUWE1 mediates K63-linked polyubiquitination of HAUSP to increase its deubiquitinase function [124]. In addition, K63-polyubiquitinated HAUSP serves as a scaffold to anchor HIF-1α, CBP, the mediator complex, and the super-elongation complex to enhance the gene transcription activity initiated by HIF-1α binding to the hypoxia response element in the promoters of HIF-1α target genes, including VEGF, Glut1, and Twist1 [124]. As HAUSP is shown to be a deubiquitinase of HIF-1α [124], the K63-linked polyubiquitination of HAUSP further enhances its ability to deubiquitinate HIF-1α. Therefore, K63-linked polyubiquitination of HAUSP improves both its deubiquitinase activity and its transcription complex scaffolding activity. These properties make HAUSP a unique HUWE1 substrate. Finally, as HIF-1α plays a major role in inducing tumor progression [125], K63-polyubiquination of HAUSP mediated by HUWE1 would contribute to the oncogenic function of HUWE1 (Fig. 5). As HUWE1 is induced by hypoxia [124], the inter-relationship between HUWE1 and HIF-1α will make HUWE1 a re-enforcing target in the complex hypoxia-induced tumorigenesis network.

### Tiam1

It has recently been shown that HUWE1 may participate in cell movement through HGF-induced RAC signaling [126]. T lymphoma invasion and metastasis inducing protein 1 (Tiam1) serves as a guanine nucleotide exchange factor (GEF) for the activation of the small GTPase, Rac [127]. *Tiam1* deficiency leads to increased apoptosis during the initiation of Ras-induced skin tumors and reduced proliferation during promotion [128]. The impaired Tiam1-mediated Rac activity leads to decreased levels of intracellular reactive oxygen speicies (ROS), which, in turn, blocks ERK phosphorylation and activation [129]. Nevertheless, the few tumors arising from *Tiam1*^−/−^ mice are more malignant and invasive compared to those from Tiam^+/+^ mice [128], raising the possibility that Tiam-Rac regulates different aspects of Ras-induced tumorigenesis. It turns out that Rac activation has dual roles in cell movement. Tiam1-Rac can enhance lamellipodia and invadopodia formation in breast cancer at a low cell density [130] but it can also promote E-cadherin-mediated adhesion in MDCKII cells [131, 132]. In accordance with the role of Tiam1 in maintaining the cell junction structure, Vaughan et al. have shown that in response to HGF, Tiam1 can be ubiquitinated at Lys-595 by HUWE1, resulting in Tiam1 degradation and disassembly of cell junctions, increasing cell migration and invasion in lung carcinoma cells [126]. HGF-induced Rac1-mediated cell migration is regulated by another HECT E3 ligase, HECT domain and Ankyrin repeat containing E3 ubiquitin-protein ligase 1 (HACE1), which degrades Rac itself [133]. Thus, ubiquitination can act as a rapid response mechanism to mitogens to ready the cells for possible movement.

### MyoD

HUWE1 is involved in myogenesis by regulating the stability of MyoD, which is one of the master transcription factors determining myoblast proliferation and myotube differentiation [134]. The transcriptional activity of MyoD is regulated at both the epigenetic level and post-translational level [135, 136]. MyoD degradation is mainly mediated by a muscle-specific E3 ligase, MAFbx/AT-1 [137], but a growing line of evidence has shown that other E3 ligases may be involved. Tom1, a homolog of HUWE1 in *S. cerevisiae*, serves as the MyoD E3 ligase and human HUWE1 in mammalian cells mediates the ubiquitination of the N-terminal residue lysines of MyoD [138]. As myogenesis is mainly regulated by fibroblast growth factor (FGF), it remains to be determined whether HUWE1 is affected by FGF, thereby influencing MyoD expression and the cell fate of myoblasts.

### Histones

HUWE1 ubiquitinates multiple histones (H1, H2A, H2B, H3, and H4) during spermatogenesis. To evolve into elongated mature forms, spermatids require degradation of a fraction of proteins, including histones. Loss of histones by HUWE1-mediated ubiquitination subsequently causes chromatin condensation into the narrow head of the mature spermatids [28]. HUWE1-mediated ubiquitination of specific histones can be detected in other physiological scenarios. HUWE1 mediates ubiquitination and degradation of histone H1.3 has been reported to regulate normal ovarian epithelial cell transformation and tumor growth [139]. H1.3 is a specific repressor for the noncoding oncogene H19 in ovarian cancer, where H1.3 overexpression occupies the H19 regulator region encompassing the imprinting control region (ICR), along with increased DNA methylation and reduced binding of the insulator protein CTCF at the ICR [140]. HUWE1 thus controls the H1.3-H19 cascade to promote ovarian tumorigenesis [139] (Fig. 2). Another histone that can be ubiquitin-modified by HUWE1 is H2AX, which is maintained at a low level in resting cells [141] but stabilized and phosphorylated to produce γH2AX at DNA double-strand breaks (DSBs) [142]. Atsumi et al. have shown that H2AX is poly-ubiquitinated by HUWE1 and subject to degradation. Upon DSBs, H2AX is dissociated from HUWE1 and enhances incorporation with chromatin regulated by SIRT6 and SNF2H [142] (Fig. 4). It would not be surprising if HUWE1 may contribute to modulation of other specific histones, although physiologic cues have not yet been found.

## Clinical relevance and therapeutic strategies

HUWE1 controls the expressions or activities of many pivotal downstream proteins. Numerous studies have shown that HUWE1 can be either oncogenic or tumor suppressing, depending on the leading substrates that it regulates in the context [see “Substrates of HUWE1”]. HUWE1 is often found over-expressed in cancers of breast, lung, prostate, colon, larynx, stomach, and uterus but under-expressed in brain tumors [43, 143]. In SHH-type medulloblastoma, high expression of HUWE1 correlates with a better survival outcome [71], indicating that HUWE1 potentially serves as a tumor suppressor and a prognostic marker in brain tumors. The small-molecule therapeutic by GDC-0449, a drug that targets the serpentine receptor Smoothened (SMO), has been used to treat a patient with advanced medulloblastoma, in which a rapid yet transient regression and an incomplete response to inhibition of the hedgehog (Hh) pathway were observed [144]. Drug resistance against GDC-0449 likely results from SMO mutation in the Hh signaling pathway [145]. Therefore, a combination therapy downregulating both SHH signaling and Atoh1 by HUWE1 should be taken into consideration for brain tumors therapy, which may be a more promising therapeutic avenue.

In tumors where HUWE1 acts as an oncogene, blockage of the enzymatic activity of HUWE1 or a direct inhibition of HUWE1 expression is useful in counteracting pathways governing cell proliferation. Being one of the well-known substrates of HUWE1, Myc provides the major pro-proliferative signaling for cancer cells and indeed, several avenues to block Myc and its signaling transduction have been designed [146–148]. Recently, a high-throughput screening of small molecules was conducted to identify two HUWE1 inhibitors, BI8622 and BI8626 [149]. Peter et al. exploited the fact that the HECT-domain of HUWE1 can autoubiquitinate and small molecules that block autoubiquitination fluorescence signals can be selected as potential inhibitors of HUWE1 in vitro [149]. Their findings show that inhibition of HUWE1 accumulates Miz1, thereby repressing Myc-activated target genes in colorectal cancer cells [149]. In accordance, in Myc-driven B cell lymphomas, HUWE1 inhibition by its specific siRNA increases p53 levels and reduces the transcriptional activity of Myc, thereby inducing apoptosis in tumors cells [88]. Thus, a direct downregulation of HUWE1 by antisense oligonucleotide (ASO) therapy delivered with nanoparticles to enhance transfection efficiency may be further applied in therapeutic intervention of cancer.

Zhang et al. have lately developed another high-throughput system-wide platform using the phage-displayed ubiquitin variants (UbV) that inhibit or activate HECT E3s [150]. They further demonstrated by structural analysis that UbV inhibitors occupy the E2-binding site whereas the UbV activators bind to a ubiquitin-binding exosite [150]. The HUWE1 inhibitors (HU.1 and HU.2) they identified reduced in vitro autoubiquitination and stabilized the protein levels of HUWE1 and c-Myc. Further application of HU.1 and HU.2 in cancer therapy needs to be tested. Interestingly, the crystal structure of the C-terminal part of HUWE1, which encompasses the catalytic domain, shows an asymmetric auto-inhibited dimer and an activation segment (residues 3843–3895) [151] (Fig. 1a). Interaction between the tumor suppressor p14ARF and the activation segment promotes oligomerization of HUWE1, therefore shifting the conformational equilibrium of HUWE1 toward the inactive state [151]. Together with Zhang’s finding [150], these data reveal that the activity of HUWE1 is largely determined by its conformation. Disruption of the proper enzymatic conformation of HUWE1 should be taken into consideration when small molecule inhibitors or peptides are developed as a novel therapeutic intervention in the future, which will fulfill the principal rule of precision medicine based on tumor types, genetic contents, and molecular/cellular presentations.

## Challenges, perspectives, and conclusion

As more findings have demonstrated the significance of post-translational modification in cancer regulation, molecular pathways which play a role in this process may serve as not only a biomarker of cancer progression but also a possible target for cancer therapy. Undoubtedly, HUWE1 is such a candidate E3 ligase that mediates ubiquitination and has shown significant implication in clinical applications. However, a precise understanding of the HUWE1 mechanism lies heavily on the identification of its substrates, which also poses technical challenges in certain aspects. First, ubiquitination is a tightly controlled, highly coordinated, and dynamic process which involves E3 ligases and deubiquitinating enzymes (DUBs) in response to environmental changes. The dynamic nature of ubiquitination is manifested by the weak physical interaction between an E3 ligase and its substrate and rapid dissociation rate. Traditional immunoprecipitation followed by mass spectrometry may only fill in a piece of the map without comprehensively identifying the substrates. Second, the fact that a single E3 ligase targets multiple substrates and a particular substrate is regulated by several E3 ligases provide significant degrees of redundancy and this web-like regulations between E3 ligases and substrates under different temporal and spatial conditions make the cellular analysis difficult [152]. Third, although the WW domains of NEDD4 family E3 ligases associate with proline rich PPxY (PY) motifs or phosphoserine/threonine residues in their substrates [153], other HECT E3 ligases, including HUWE1, have not been shown to bind specifically to a certain consensus motif. Recently, systemic and quantitative approaches using monoclonal antibodies that recognize diglycine (diGly)-isopeptide have been utilized to characterize ubiquitin-modified proteome [154, 155]. Most of the identified substrates of HUWE1 discovered so far have K48-linkage or K63-linkage polyubiquitins (Table 1). Michel et al. have used K6-linkage-specific “affimer” to identify HUWE1 as a main E3 ligase for this chain type [42]. These bettered “ubiquitome” techniques can enhance the breadth and depth of HUWE1 studies. Further characterization on the signaling pathways that modulate the activity or the expression of HUWE1 as well as the chain types of its downstream targets may assist us to understand the underlying mechanism of tumorigenesis and metastasis. In sum, these molecular and cellular researches can later be applied to translational studies as new therapeutic approaches for tumor treatment.

**Table 1:** Substrates of HUWE1-mediated ubiquitination

Targets	Ubiquitination Type and Linkage	Ubiquitination Effects	Functional Alteration by ubiquitination	References
c-Myc	K63-linked poly-ubiquitination	c-Myc transactivation	Increased cell proliferation	[43]
N-Myc	K48-linked poly-ubiquitination	Degradation	Dysregulated neural differentiation; Perturbed HSC self-renewal	[60–62]
Dvl	K63-linked poly-ubiquitination	Inhibit Dvl multimerization	Negative feedback loop to Wnt signaling	[63]
Atoh1	Poly-ubiquitination	Phosphorylation-dependent degradation	Increased neuronal differentiation	[71]
Mcl-1	Poly-ubiquitination	Degradation	Increased DNA-damage induced apoptosis; Increased hepatocyte apoptosis	[77]
p53	Poly-ubiquitination	Degradation	Reduced cell growth arrest; Increased anti-apoptosis	[87]
HDAC2	Poly-ubiquitination	Degradation	Apoptosis	[92]
Miz1; c-Myc/Miz1	K48-linked poly-ubiquitination	Degradation	JNK activation and apoptosis; Ras signaling suppression;	[30, 102]
Cdc6	Poly-ubiquitination	Degradation	Released from chromatin; Inhibition of preRC assembly	[103]
BRCA1	Poly-ubiquitination	Degradation	Reduced DNA repair	[116, 117]
Pol β	Mono-ubiquitination	Degradation	Decreased DNA repair	[39]
Pol λ	Mono-ubiquitination	Degradation	Decreased DNA repair; Reduced chromatin binding	[40]
HAUSP	K63-linked poly-ubiquitination	Enhanced HAUSP deubiquitase activity; enhanced transactivation	EMT, chromatin modification	[124]
TIAM1	Poly-ubiquitination	Degradation	Enhanced migration, invasion	[126]
MyoD	Poly-ubiquitination	Degradation	Myogenesis	[138]
H1.3	Poly-ubiquitination	Degradation	Ovarian epithelial cell transformation; Tumor growth	[139]
H2AX	Poly-ubiquitination	Degradation	Decreased DNA repair	[142]

## Abbreviations

BER: Base excision repair
CHIP: Carboxyl terminus of Hsc70 interacting protein
DSBs: Double-strand breaks
Dvl: Dishevelled
GNPs: Granule neuron progenitors
HDACis: HDAC inhibitors
HDACs: Histone deacetylases
HECT: Homologs to the E6-AP carboxyl terminus
HR: Homologous recombination
HSC: Hematopoietic stem cells
HUWE1: HECT, UBA and WWE domain containing 1, E3 ubiquitin protein ligase
MB: Medulloblastoma
NHEJ: Non-homologous end joining
preRC: Prereplication complex
SCF: Skp1/Cul1/F-box
SHH: Sonic hedgehog
UBA: Ubiquitin-associated
